# Dental Quacks

**Published:** 1858-09

**Authors:** 


"SPEAK THE SPEECH I PRAY YOU AS I PRONOUNCE IT TO YOU."—*Shakspere.*

The great poet of the people said that "this world is a stage and all the men and women are players." The object of the drama is to hold the curtain up to nature, and exhibit at a glance the varied phases of the act, so that the beholder may draw a distinction between the actors, and form, by comparison, a conclusion as to the merit of the scene.

The ultimate object is to sustain morality and suppress vice.

To do this it is necessary to sustain by competent actors the several parts, so near to the original, that for the time being the beholder will become in feeling one of the actors.

To show the beauty of morality, vice must be well represented, and in allotting the parts to the actors, great care and consideration is necessary to choose actors well fitted to the peculiar cast of the piece.

A good tragic actor seldom plays comedy well, neither do we expect one accustomed to play the moral, to make a finished villian. Thus it is in the imitative drama, the actors perform the part assigned to them, not being responsible for the results of the other parts, hence the failures. But in the real drama of life we choose the parts for ourselves, and are to a certain extent responsible for the whole play.

If moral principles govern our feelings we are apt to select a corresponding character. If vice predominates, though we may try to imitate the moral, sooner or later the real character comes out.

In this drama of life, there are scenes where the dentist makes his entrance. His part as arranged is one of strictly moral cast, but oftentimes it is chosen by unworthy actors, and so rendered as to become one worthy the lowest and most degraded performer on the stage. And was it, as in false drama, that the actor only was to be affected by bad playing, all would be well, but in our real drama the audience is alone to be abused and dishonored.

This game of bad playing among dentists is becoming so common, and having so bad a tendency, that it may not be amiss to give a gentle hint to the actors, that they may try to reform. And if hints prove not sufficient, more stringent measures may be called into action, whereby the play may not be interrupted, and the actors made to play their parts alone deprived of their masks.

At the present time we propose to extend our criticism to only one class of players, namely, the undergraduates and graduates of our dental colleges. And while we would protect the innocent, we mean that the guilty may not go unwhipt of justice.

The evil of which we shall speak has existed for some time, but from feelings of delicacy toward the profession, the subject has been allowed to pass until tolerance has ceased to be a virtue.

The practice of falsely using the names of our dental colleges in order to increase business has been carried on to a great extent by unprincipled villians, until the college faculties have found it necessary to insert in their annual circulars all of the names of their graduates for the whole time the college has been in existence. But these circulars reach only the profession, while those who are to be most affected are still ignorant of the fraud practiced upon them. And to such an extent is this carried on in places remote from the colleges, that the name of Dental College is but another name for humbug.

But this for the most part has been done by those who have never even been inside of a dental college, and over whom we have little if any control, and out of reach of any remedy to suppress the evil.

But those whom we wish more particularly to deal with at present, is the graduated quack and the one who has attended one course of lectures, and not a graduate. Of the latter very many are pretending to be graduates, and some have the boldness and audacity to attach the title to their names. As a general thing these persons are in places where but little of their doings reach the city. Hence they do with impunity their deeds of rascality.

And taking their words for it, they are posted in every branch of their profession, and without hesitation they challenge competition with the best in the profession, knowing that their egotism is confined to the immediate vicinity in which they reside.

We could give some striking instances of the quackery of this class, and may hereafter bring before the profession, some choice and interesting specimens of their cards and advertisements, but space at present forbids.

I will now notice the graduated quack and will illustrate by only two out of several cases on hand.

My first case will show a sample of gratitude towards the alma mater.

Dr. ——, who is a regular graduate is often heard from through the daily papers recounting his unbounded success in practice, and informing the community that he is ready to serve them with his splended artificial fixture, invented and inserted alone by himself, and one which is bound to work an extensive revolution in the department of practice. He also informs them that he can perform other wonders and cures that the balance of the profession are entirely ignorant of.

And though publicly opposed to secret practice he is daily vaunting his skill in that respect.

He uses the title conferred upon him by the college, but is often heard to abuse the institution, and also some of the faculty who belong to his own profession, calling them quacks and villains. And the very department that showered the greatest honors upon him, comes in for the greatest share of his abuse and maledictions.

Hereafter we may give to the profession some of his choice literature as found in the daily papers, with the paid editorials.

But showing this actual case as a type of several of his class now on the black list, we shall leave him while we show up one of his twin brothers in another act of our drama, giving a specimen of dental advertising.

The following we find in a country newspaper under the date of June 17, 1858.

"DENTAL PARTNERSHIP.

"Dr. J——— has united in partnership with Dr. C——— late of Cincinnati, in the practice of dental surgery.

"Dr. C——— comes highly recommended as a skillful operator, having graduated at the Cincinnati Dental College and had an experience of eight years practice under the most favorable circumstances for becoming thoroughly posted in his profession.

"Dr. J's superior qualifications as a dentist are too well known in this community to need comment.

"Together these two gentlemen, we can safely guarantee, will give general satisfaction to the public.

"Persons desiring their services, at night, will find one of the firm at the office."

The above card reads very well, and when endorsed and guaranteed by the editor, is one that should give the public confidence in the dental firm.

But supposing that the editor is entirely innocent of the part he is playing and sufficiently well paid to keep his conscience at ease, let us for a moment glance at the history of one other of the performers, namely that of Dr. C.

Presuming a part of his story to be true, we would ask him, when and where he got his "*eight years of practice under the most favorable circumstances*" And while we leave him to answer our question, we venture this assertion, that on the first of November, 1856, he was employed in a sash and door factory in one of the interior towns of Ohio,—previous to which he had never performed an operation in the mouth, or mounted an artificial tooth in any manner. Neither had he any qualifications by medical or dental study under either a medical or dental practitioner.

His first entrance into the profession was to attend the lectures at the Ohio Dental College during the session of

1856–7. He spent a portion of the summer of 1857, under an instructor,—attended the lectures in the college during the session of 1857–8, spent the time from close of session to about the first of June in the college infirmary, when we find him with costume all changed on the 17th of the month ready for another act in our drama of professional life.

The bell has rung, the curtain risen and the actor stands before his audience ready to receive their smiles.

The question now arises why does the Doctor use such strong language in bringing himself before the public. Did he suppose that his cards would not reach the eye of others who were posted in the facts, and that he would thereby do no wrong to the profession?

How does he construe his Diploma? How far does it license him to go in misrepresenting his standing in the profession to the public?

Does it give him liberty to extend weeks of time into years, and make a tyro into a skillful operator?

If such is their power, then diplomas are procured entirely too cheap, for much is rendered where little is paid. And if they have such force, it would be well for many who are now destitute of them to use great exertion to obtain them, for they would be the "open sesame" to wealth and honor. But let us inquire what the diploma does guarantee to the possessor, and how far it is a passport to public confidence. Also, what are the obligations the graduate lays himself under to the college, when he receives this mark of favor.

In the first place, the diploma is a certificate that the bearer has passed through a satisfactory examination on certain subjects of theory, preparatory to entering on the practical part of the profession. It testifies that a firm and scientific foundation has been laid, whereon the student may erect his superstructure. Also that the road has been pointed out to him and guides set by competent and honest directors, in which if he travels, the goal of eminence is sure to be reached by a direct and honorable course. It is a recommendation to the public that the bearer will, by his manly bearing and honest dealings, so serve them as to gain and merit their confidence and patronage.

It does not testify to the public that he is a skillful operator, or that he has served any specified time in the profession, either alone or with any other operator. But the intent is to show his proficiency in the theoretical, rather than the practical, for all good operators well know that it takes years of successful practice to become a skillful operator.

But it is presumed that after the plans are all drawn and the foundation well laid, that the diligent student will in time erect a structure beautiful in all its proportions and creditable both to himself and his alma mater.

When the diploma is given, it is understood by those giving it, that the receiver pledges himself to use all honorable means to become a skllful operator,—an honor to the college and to the profession of which he becomes a member. Under no other considerations would it be granted.

But if the course was pursued by all of the graduates that has been by a few, how soon would our colleges fall into disrepute and their diplomas be worthless.

But fortunately the great majority of the graduates have proved an honor to themselves and to the college that they received their honors from.

Having set their standards of honor and qualifications high, they are sure to reach the highest point of their ambition.

And by keeping in mind and carrying out the good precepts taught them on leaving college, they show their gratitude to their alma mater by making themselves worthy the honors she bestowed upon them.

But where will the other class, those who dishonor her, be found? Echo answers, where? For the present we leave our actors, in hopes that ere too late, they will seek a reformation, but if their course is still pursued, their names will follow in quick succession, and reach them and their patrons through the same medium that they choose for their own emolument, with such facts as will clearly show both sides of the question instead of one.

It were well if the evil were confined to the graduates of one college, but unfortunately each one can claim its share of these false actors.

I know of but one remedy, which is for every lover of our profession to watch over and guard our dental colleges, and when any of their graduates prove recreant to principles of honor, to boldly expose them to the world.

In this way dental colleges will be sustained, and their diplomas be what they should be, a certain passport to favor and patronage, which is the wish of one of the ALUMNI.

